# MicroRNA Profiling during Cardiomyocyte-Specific Differentiation of Murine Embryonic Stem Cells Based on Two Different miRNA Array Platforms

**DOI:** 10.1371/journal.pone.0025809

**Published:** 2011-10-03

**Authors:** Lin Gan, Silke Schwengberg, Bernd Denecke

**Affiliations:** 1 Interdisciplinary Centre for Clinical Research (IZKF) Aachen, RWTH Aachen University, Aachen, Germany; 2 Axiogenesis AG, Köln, Germany; University of Frankfurt - University Hospital Frankfurt, Germany

## Abstract

MicroRNA (miRNA) plays a critical role in a wide variety of biological processes. Profiling miRNA expression during differentiation of embryonic stem cells will help to understand the regulation pathway of differentiation, which in turn may elucidate disease mechanisms. The identified miRNAs could then serve as a new group of possible therapeutic targets. In the present paper, miRNA expression profiles were determined during cardiomyocyte-specific differentiation and maturation of murine embryonic stem (ES) cells. For this purpose a homogeneous cardiomyocyte population was generated from a transgenic murine ES cell line. Two high throughput array platforms (Affymetrix and Febit) were used for miRNA profiling in order to compare the effect of the platforms on miRNA profiling as well as to increase the validity of target miRNA identification. Four time points (i.e. day 0, day 12, day 19 and day 26) were chosen for the miRNA profiling study, which corresponded to different stages during cardiomyocyte-specific differentiation and maturation. Fifty platform and pre-processing method-independent miRNAs were identified as being regulated during the differentiation and maturation processes. The identification of these miRNAs is an important step for characterizing and understanding the events involved in cardiomyocyte-specific differentiation of ES cells and may also highlight candidate target molecules for therapeutic purposes.

## Introduction

Embryonic stem cells (ES cells) are pluripotent cell lines generated from the inner cell mass of blastocysts [1]. The spectrum applications of ES cells is diverse and includes drug discovery, high-throughput toxicology assays, regenerative medicine and embryology [2], [3]. Primary cells, such as hepatocytes or cardiomyocytes cannot be maintained in culture for long periods of time [4], [5], [6]. Furthermore, there is a strong donor-dependent variability [7]. This is not a problem for immortalized cell lines, however, they have been shown to be genetically unstable and do not fully emulate the features of their primary cell counterparts. In contrast, ES cells have an almost unlimited self-renewal capacity in their undifferentiated state and the ability to differentiate into fully mature cells of all cell types of the three embryonic germ layers [8], [9]. Thus, ES cells may constitute a unique source of differentiated cell types and, as such, the regulation of their differentiation pathways is under intensive study.

The phenotype of a cell is controlled by gene regulation, which is the basis for cell differentiation, morphogenesis and the adaptability of cells [10], [11]. Modification of gene expression can occur at different levels. Apart from epigenetic mechanisms (cytosine methylation, histone acetylation), regulation can be observed at the level of transcription initiation (transcription factors), heteronucleic transcript processing (RNA splicing), mRNA transport from the nucleus into the cytoplasm (nucleocytoplasmatic transport factors, e.g. exportin-5), translation and post-translational modifications [12], [13]. It has recently become evident that the previously widely ignored non-protein coding genes play an important role in the control of gene expression. MicroRNAs (miRNA) have become one of the most important regulation factors and an understanding of their expression and influence during ES cell differentiation will help in the elucidation of the whole process [14], [15]. In contrast to single analysis, miRNA expression can be profiled in a more time- and cost-effective manner by applying microarray technology [16]. Results from different microarray platforms are not always very well correlated even for same samples due to differences in design, manufacturing, hybridization condition, and label/detection methods. In practice, microarray data are often verified using other techniques, such as RT-qPCR, but in principle, the result from one high throughput platform could also be verified with another independent high throughput platform.

In the present study, both Affymetrix miRNA 1.0 arrays and Febit Biochips miRNA arrays were used in profiling miRNA expression during controlled differentiation of mouse ES cells to cardiomyocytes. Affymetrix miRNA 1.0 arrays cover 610 mouse miRNAs and Febit miRNA arrays cover 719 miRNAs, 609 of which are common to both platforms. Febit arrays provide additional information of 110 miRNA for mouse, while Affymetrix arrays have one miRNA (miR699) that is not covered by the Febit array.

Affymetrix applies photolithography *in-situ* oligo synthesis technology, which enables very high feature numbers on chips [17]. Oligonucleotides with a maximum length of 25mer were synthesized directly on chip. The GeneChip® miRNA Array covers miRNAs from 71 organisms on a single array including human, mouse and rat. The GeneChip miRNA Array also includes human small nucleolar RNAs (snoRNAs and scaRNAs), which are short, non-translated RNAs that play a role in the processing of ribosomal RNAs following transcription. snoRNAs have also been implicated in the regulation of alternative splicing. Samples were labeled with biotin before hybridization and stained with fluorescent labeled streptavidin after hybridization.

In contrast to Affymetrix technology, Febit Biochips were produced using light-activated *in-situ* oligonucleotide synthesis by means of a digital micromirror device. The probes are designed as the reverse complements of all major mature miRNAs and the mature sequences as published in the Sanger miRBase release (version 14.0 September 2009, see http://microrna.sanger.ac.uk/sequences/index.shtml) for mus musculus. Additional nucleotides are bound on the 5′-end of each capture oligonucleotide necessary for the on-chip labeling technology MPEA (Microfluidic Primer Extension Assay). This special procedure extends and labels perfectly hybridized probes directly on the chip. With this method, noise resulting from mismatch probes is expected to be reduced.

Due to the great differences in probe design, array production and hybridization techniques used to generate Affymetrix- and Febit-miRNA arrays, they can both be considered as two entirely independent platforms.

To investigate the processes of cardiac differentiation of mouse ES cells, we are interested in miRNA profiling during cardiomyocyte-specific differentiation and maturation events. To date, considerable effort has been made in the establishment of reproducible protocols to control stem cell growth and differentiation. Regardless of the large variety of differentiation protocols that have been established, it is difficult to generate a uniform population of cells [18], [19]. In order to collect valid results to understand a specific differentiation process it is particularly important to have homogeneous, synchronized and clonal cell populations available for the undifferentiated as well as for the differentiated cell type. For this reason, a transgenic mouse ES cell clone was used in our experiments. This clone is derived from D3 ES cells [20] that had been stably transfected with DNA constructs allowing the expression of puromycin resistance (PAC) gene and enhanced green fluorescent protein (EGFP) reporter gene under the control of mouse cardiac-specific ã-myosin heavy chain (ã-MHC) promoter. Through induced differentiation and selection a highly pure cardiomyocyte cell population (>99.9%) can be produced. Such Cor.At® cardiomyocytes are fully functional cardiomyocytes which contract spontaneously and rhythmically and express cardiac-specific genes including those relevant for cardiocyte ion channels [21], [22]. When transplanted into a cryoinfarcted area of the mouse heart, such donor cells fully integrate into the host cardiac tissue and restore functionality [22]. With the transgenic ES cell clone as reference, Cor.At® cardiomyocytes are an ideal model for cardiac-specific differentiation and maturation research.

In the present study, Affymetrix miRNA gene arrays and Febit Geniom Biochips miRNA arrays have been used in parallel to profile miRNAs during the differentiation of mouse ES cells to cardiomyocytes. The comparison of undifferentiated ES cells with highly pure cardiomyocytes derived from the same stem cell clone enables the examination of changes in miRNA expression during differentiation and maturation under defined conditions. On one hand, the data can help in understanding the impact of platforms in miRNA profiling and, on the other hand, assist in the verification of miRNA profiling data obtained with the platforms.

## Results

### *In vitro* differentiation and maturation of ES cells to cardiomyocytes

**Figure 1:**
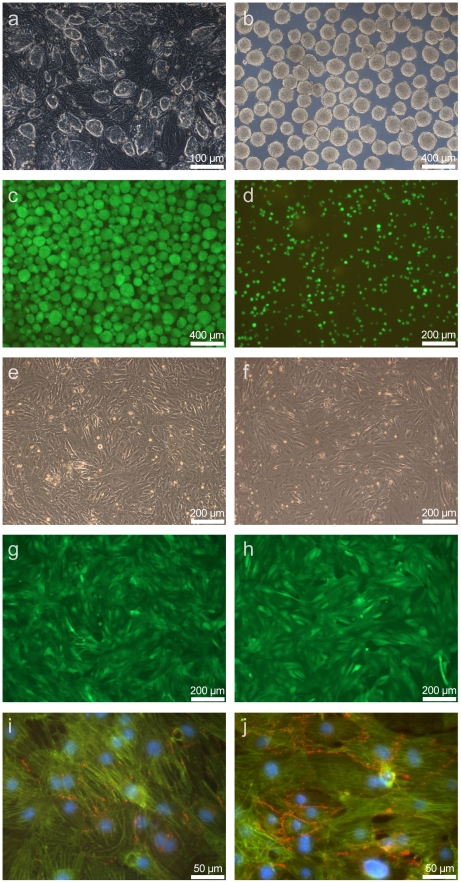
Differentiation and maturation of undifferentiated ES cells to Cor.At® cardiomyocytes. a) Undifferentiated mouse ES cells (clone αPIG44) on feeder cells. b) Mouse ES cells aggregated into EBs at day 3 after initiation of differentiation. c) Mouse ES cell derived Cor.At® cardiomyocytes after 12 days of differentiation and 3 days of puromycin treatment, before dissociation. d) Mouse ES cell derived Cor.At® cardiomyocytes after 12 days of differentiation and 3 days of puromycin treatment, after dissociation (probe Cor.At® cardiomyocytes 12 days). e,g,i) Cor.At® cardiomyocytes after additional 7 days of culture (probe Cor.At® cardiomyocytes 19 days): e) transmission, g) same region EGFP fluorescense indicating differentiation to cardiomyocytes, i) overlay of immunostainings: blue = nucleus staining with DAPI, green = α-actinin (structured) and GFP, red = connexin 43. f, h, j) Cor.At® cardiomyocytes after additional 7 days of culture (probe CorAt 26 days): e) transmission, g) same region GFP fluorescense indicating differentiation to cardiomyocytes, i) overlay of immunostainings: blue = nucleus staining with DAPI, green = α-actinin (structured) and GFP, red = connexin 43.

The use of pure ES cell lineages is a prerequisite to be able to make reliable statements about changes in miRNA expression during differentiation and maturation to cardiomyocytes. A transgenic mouse ES cells clone (αPIG44) has been used which harbors a genetic construct with a puromycin resistance cassette and an EGFP reporter under the control of the cardiac α-MHC promoter. These ES cells can be propagated continuously on feeder cells in an undifferentiated state (Figure 1a), avoiding the variability often observed by using different lots of primary cells. This pure population of undifferentiated ES cells can be compared to Cor.At® cardiomyocytes originating from the same clone by inducing differentiation via the formation of embryoid bodies (Figure 1b). An essential advantage in obtaining reliable results is that following the differentiation protocol, cells that have not differentiated to cardiomyocytes can be killed with the selection agent puromycin. This prevents contaminations with RNAs from other cell types. Differentiation to Cor.At® cardiomyocytes can be easily monitored by the presence of the EGFP reporter gene that is selectively expressed by cardiomyocytes. Three days after puromycin-mediated selection for Cor.At® cardiomyocytes, the cells in the cardiobodies demonstrate EGFP reporter activity (Figure 1c) which can also be seen after plating as a single cell suspension (Figure 1d). During further culture, the cells become larger and cell-cell contacts are established (Figure 1e, g). Striated α-actinin structures are formed by day 19 and gap-junctions between the cells become visible (connexin-43 staining) (Figure 1i). The detection of which is increased by day 26 (Figure 1f, h, j). Cor.At® cardiomyocytes display a normal, stage-specific electrophysiological phenotype that matches their *in vivo* counterparts, as described in the literature [21], [23], [24] (data not shown).

### The expression levels of miRNAs are distributed differently on the two platforms

The profiling of miRNAs during differentiation and maturation can provide a good approach to understand the process of cardiomyocyte differentiation. For this purpose, miRNA samples were analyzed from undifferentiated ES cells (day 0) and from cells at different time points after differentiation to Cor.At® cardiomyocytes (i.e. days 12, 19, and 26). To authenticate the information for miRNA changes, 2 different array platforms were chosen for the analyses. To exclude normalization method dependent effects, raw data of both data sets was analyzed using robust multi-array analysis (RMA) as well as with variance stabilization normalization (VSN). Since the miRNA platforms used were different in the context of the oligos on the array as well as the method used for probe-labeling, it could be assumed that the data obtained independently from each of the platforms and their respective normalization methods was highly valid.

Firstly, the number of expressed (present) miRNAs was determinded for the undifferentiated ES cells (day 0) and for the different time points after differentiation (i.e. days 12, 19, and 26). A stable number of miRNAs was observed from the analysis using the Affymetrix platform (Table 1), while the number of miRNAs increased significantly from time point day 0 to day 26 using the Febit platform. A detailed list of miRNAs detected is shown in table S1.

**Table 1:** Number of present miRNAs at different stages (day 0, day 12, day 19 and day 26) during cardiomyocyte specific differentiation and maturation.

	day 0	day 12	day 10	day 26
**Affymetrix**	318	366	323	346
**Febit**	71	184	211	343

**Figure 2:**
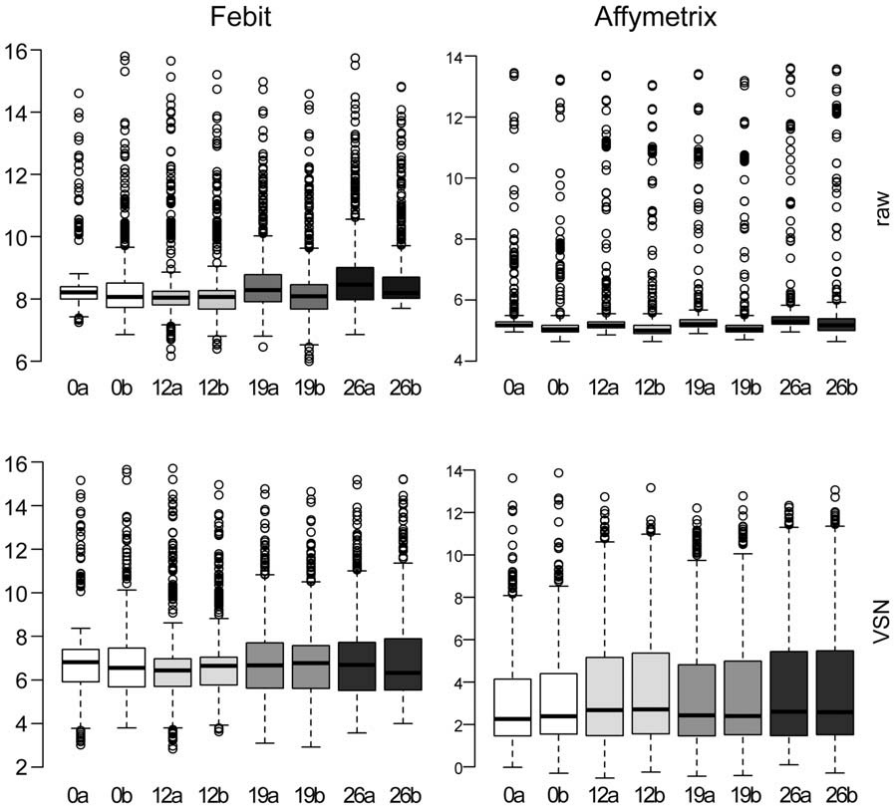
Boxplots of raw and pre-processed miRNA microarray data. Upper part left: boxplot of the raw log_2_ intensities of Febit miRNA arrays, upper part right: boxplot of the raw log_2_ intensities of Affymetrix miRNA arrays, lower part left: VSN normalized Febit miRNA array data, and lower part right: VSN normalized Affymetrix miRNA array data. X-axis shows sample names, Y-axis is in log_2_ arbitrary units. The black bar represents the median of each distribution. The open circles represent the outliers.

To get an impression of the degree of similarity between the results obtained by the two independent miRNA array platforms the data has been presented as box-plots (Figure 2). Using this kind of illustration, the degree of dispersion (spread) and skewness of the data (and outliers) has been compared. In the upper part, the raw data from Affymetrix and Febit have been plotted. The median of the samples varied slightly as well as the distances between 25^th^ and 75^th^ percentile values. The variances were greater in the Febit data. However, no outlier could be identified between the samples. The data after VSN normalization is depicted in the lower part of figure 2. The boxes containing the 25^th^ to 75^th^ percentile have been extended, and the variance of medians reduced. Data generated from the Affymetrix platform demonstrated a more even distribution than that from the Febit platform.

### Samples are well clustered according to differentiation and maturation stages

**Figure 3:**
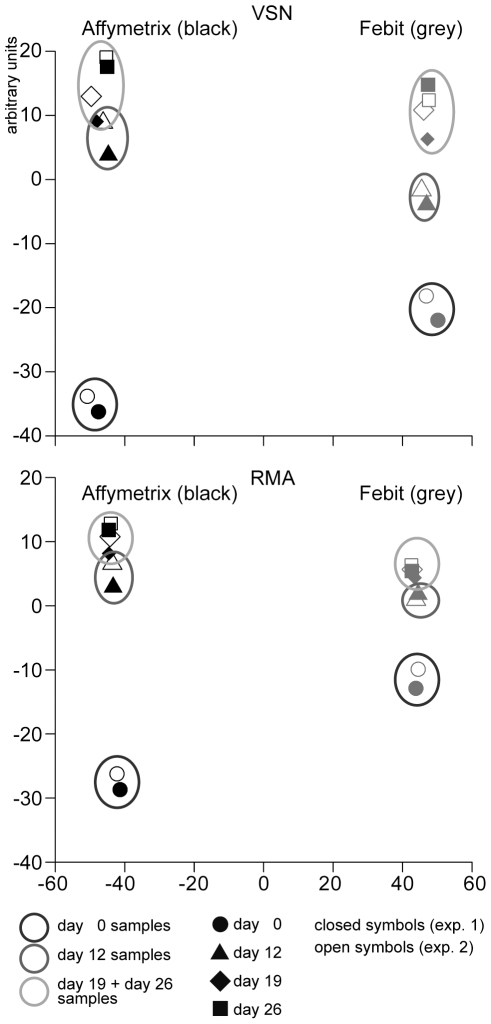
Principle component analysis (PCA) plots of microarray data. Upper part: VSN normalized microarray data from Affymetrix platform (black) and Febit platform (grey). Lower part: RMA normalized data from Affymetrix platform (black) and Febit platform (grey). Circles indicate day 0, triangles day 12, rhombuses day 19, and squares day 26. Closed symbols and open symbols represent two replicate samples from the corresponding time points after differentiation.

To abstract the relationship of the data, principle component analyses (PCA) were made for data from both platforms after RMA or VSN normalization. The first 2 components have been plotted in figure 3. The upper part of figure 3 illustrates the PCA plot of data after VSN normalization. The lower part shows PCA plot data after RMA normalization. Data points from duplicate samples were located together in both plots (circles around duplicates), indicating that both biological replica scarcely showed any difference in their variations. Data points were most clearly separated in the first component axis by platform origin, independent of the normalization method used (Figure 3). This indicated that the results showed platform-dependent differences. In the second component axis, data were separated according to differentiation and maturation stages. Day 0 (undifferentiated ES cells) samples were, as expected, far apart from the Cor.At® cardiomyocytes samples (days 12, 19, and 26). The distance from day 0 to other time points was greater for Affymetrix data than for Febit data, suggesting that Affymetrix platform was more sensitive in miRNA profiling. Day 19 and day 26 data were not very well separated. This phenomenon was more obvious on the Febit platform, suggesting that day 19 and day 26 samples were similar in miRNA expression profile.

### miRNAs are differentially expressed during cardiac differentiation

**Figure 4:**
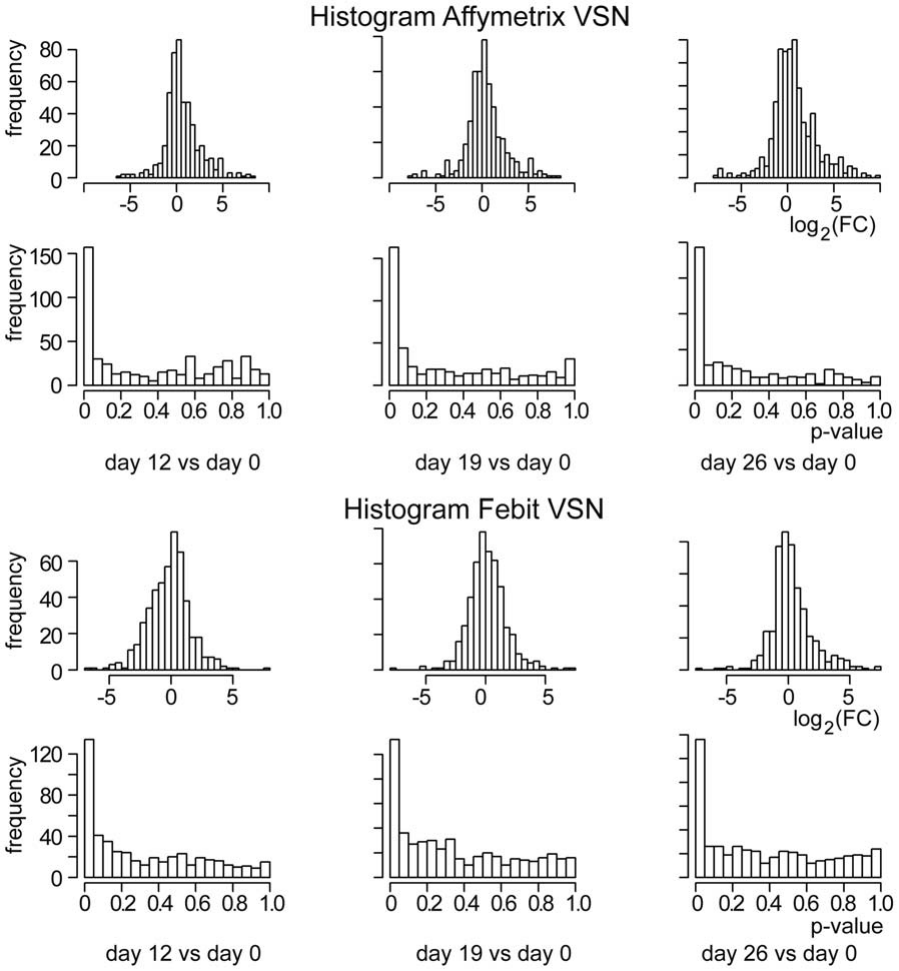
Comparison of microarray data dependent on platform used. Histograms of the log_2_ fold changes and adjusted p-values of comparisons between differentiated Cor.At® cardiomyocytes and undifferentiated ES cells for microarray data from Affymetrix (upper part) and Febit (lower part). The comparisons are day 12 vs. day 0, day 19 vs. day 0 and day 26 vs. day 0 (from left to right).

To discover the role of miRNA in cardiac differentiation and maturation, linear models were fitted for miRNA expression data with LIMMA package from Bioconductor. Three comparisons were made to analyze miRNA regulation during cardiomyocyte-specific differentiation and maturation: day 12 vs. day 0, day 19 vs. day 0 and day 26 vs. day 0. The results showed effects of platforms as well as of processing methods. Histograms of expression differences and p-values in the 3 comparisons are shown in figure 4. Results from the Affymetrix platform showed a higher number of miRNAs with little regulation compared to results obtained with the Febit platform. There were also more miRNAs with low p-values after statistical analyses for data from the Affymetrix platform.

**Figure 5:**
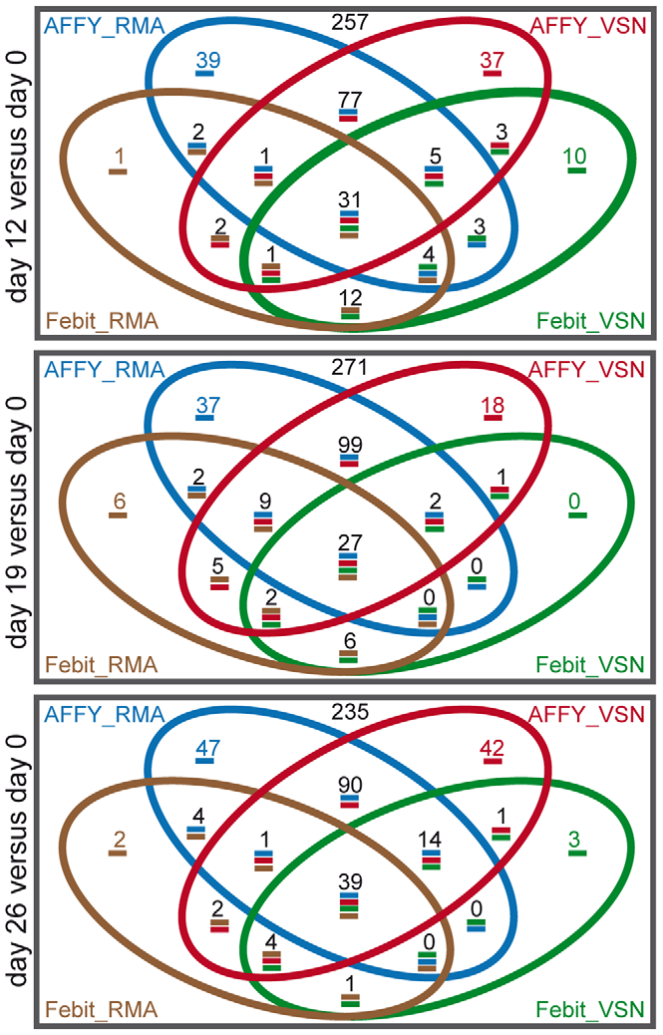
Range of overlap depends on platform and pre-processing method. Venn diagram of regulated miRNAs detected by comparing differentiated Cor.At® cardiomyocytes to undifferentiated ES cells (day 0): (upper part) in comparison day 12 vs. day 0, (middle part) in comparison day 19 vs. day 0, (lower part) in comparison day 26 vs. day 0. Blue: Affymetrix data with RMA normalization, red: Affymetrix data with VSN normalization, brown: Febit data with RMA normalization, green: Febit data with VSN normalization.

The miRNAs that were differentially expressed in a significant manner have been illustrated in Venn diagrams. The results from day 12 versus day 0, day 19 versus day 0 and day 26 versus day 0, respectively, are represented in independent Venn diagrams (Figure 5). The 4 circles in each Venn diagram represent significantly regulated miRNAs detected under 4 combinations of platform and normalization methods: Affymetrix with RMA (blue), Affymetrix with VSN (red), Febit with RMA (brown), and Febit with VSN (green). There was a good miRNA overlap between the 2 platforms as well as between the 2 normalization methods. In the comparison day 12 to day 0, 31 miRNAs were identified as regulated under all four circumstances mentioned above. 27 miRNAs and 39 miRNAs were detected for the same principle in comparisons of day 19 to day 0 and day 26 to day 0. The Affymetrix platform detected far more miRNAs than the Febit platform. For example, in the comparison of day 12 to day 0, beside the 31 platform and pre-processing method independently regulated miRNAs, there were 77 pre-processing method independent regulated miRNAs detected only from the Affymetrix platform. This number was 12 for the Febit platform. In the other comparisons (day 19 to day 0 and day 26 to day 0) similar results are shown (99 vs. 6 and 90 vs. 1, respectively).

To elucidate the overlapping results as well as the platform-dependent discrepancies, 6 selected miRNAs were analyzed by RT-qPCR assays as a third independent method. Two miRNAs (miR-1 and miR-292-3p) represented overlapping results from Affymetrix and Febit miRNA platforms. miR-295* represented those miRNAs only being identified as being regulated by the Affymetrix platform. miR-208a was an example for miRNAs which were detected as being regulated only by the Febit platform. miR-501-3p was selected on behalf of the group of miRNAs which gave controversial results with the two platforms, while miR-715 represented those miRNAs not detected as being regulated by either platform.

**Figure 6:**
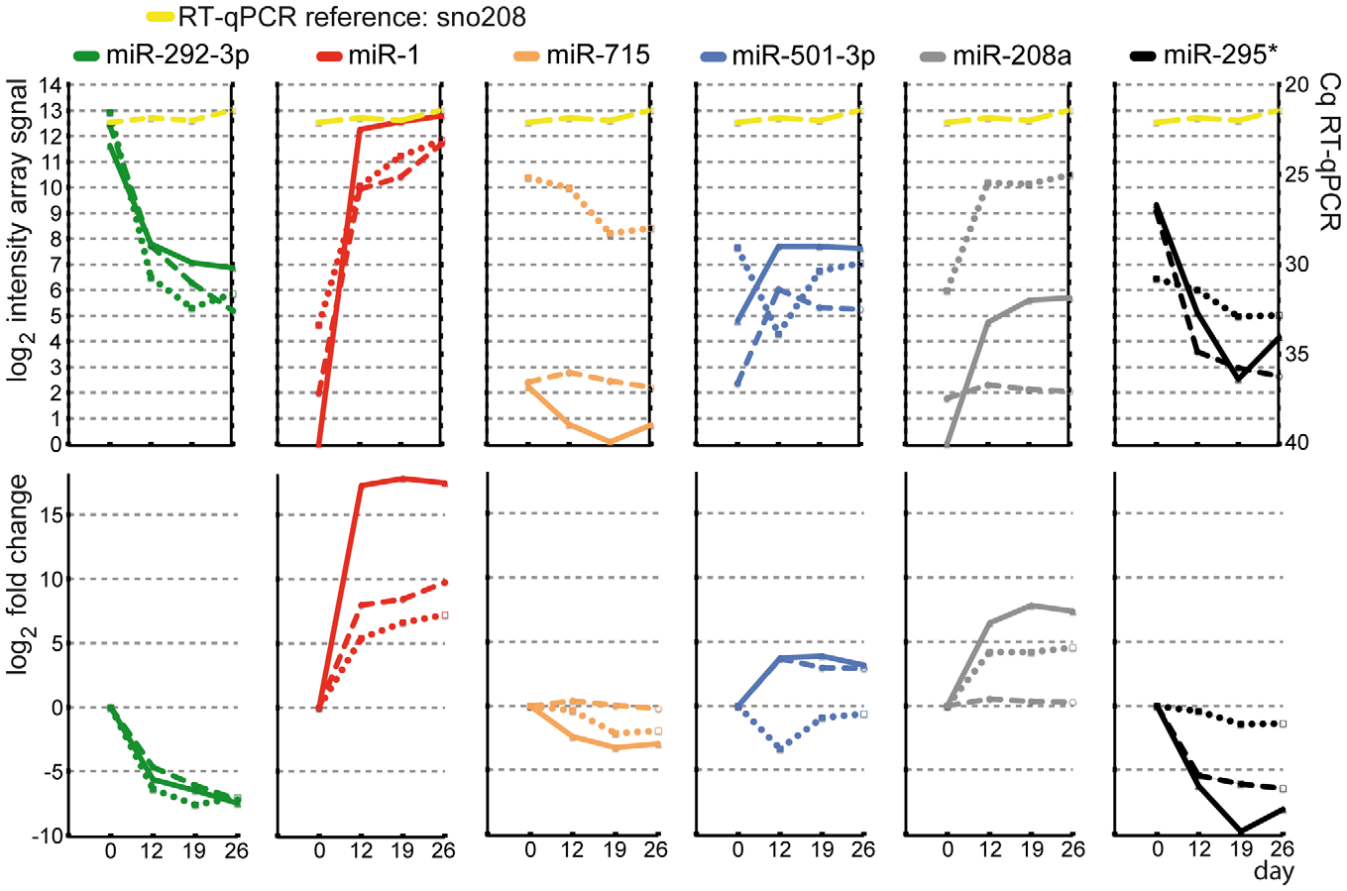
Evaluation of Array results for 6 selected miRNAs by RT-qPCR miRNA assays. RT-qPCR of selected miRNAs at day 0, day 12, day 19 and day 26 in comparison to Affymetrix and Febit array results are shown. Upper part: The log_2_ intensities of the samples analyzed by arrays (left scale) and the quantitative cycles Cq detected by RT-qPCR miRNA assays (right scale) are illustrated. Lower part: Log_2_ fold change of the amount of miRNA at the day indicated in comparison to day 0. Solid lines represent results from RT-qPCR miRNA assays; dashed lines represent results for Affymetrix array measurements; dotted lines represent results for Febit array measurements; dashed-dotted lines represent the Cq values of the RT-qPCR reference small nucleolar RNA snoRNA 202.

The RT-qPCR Cq values of these 6 miRNAs are illustrated in figure 6, and have been overlaid with their intensity values on the Affymetrix and Febit platforms. For miR-1 and miR-292-3P, results were consistent for both miRNA array platforms and the RT-qPCR data verified these results. This was also true for miR-715, which showed no change in expression with all three methods, although the absolute intensity from Affymetrix data matched better with the Cq value of RT-qPCR. For the miRNAs with controversial results from Affymetrix and Febit platforms, the RT-qPCR shared results partially with Affymetrix and partially with Febit. For example, RT-qPCR results were closer to Affymetrix results for miR-501-3p, which showed a clear up-regulation at day 12. This was also the case for miR-295*, for which both Affymetrix and RT-qPCR detected a down-regulation during the differentiation process. However, for miR-208a the RT-qPCR result was closer to the Febit result, which detected an up-regulation (whereas Affymetrix did not).

We selected the miRNAs, which were identified as being regulated under all four circumstances as platform- and analysis-independent results. All miRNAs that were identified in the comparisons between undifferentiated ES cells and Cor.At® cardiomyocytes at maturation timepoints (days 12, 19, and 26) resulted in a pool of 50 miRNAs. In the subsequent study, we focused on these 50 miRNAs.

**Figure 7:**
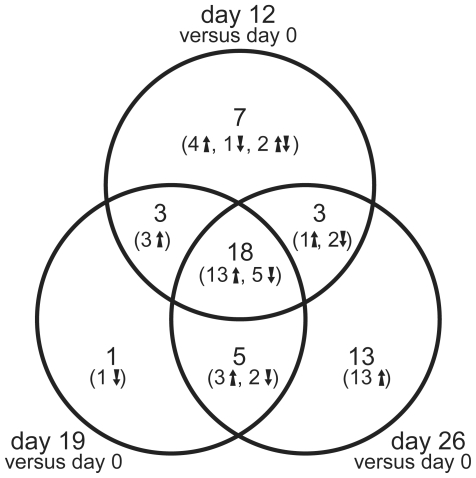
Venn diagram of the 50 platform and pre-processing methods independent regulated miRNAs. The number of up/down regulated miRNAs at different maturation stages (day 12, day 19 and day 26) compared to undifferentiated ES cells were denoted with arrows up/down.

A closer inspection of the 50 miRNAs that were regulated in a platform- and normalization-independent manner revealed that 18 of these miRNAs were regulated throughout the total differentiation and maturation process. Among these 18 miRNAs, 13 were identified as being up regulated while 5 were down regulated. There were 7 miRNAs (4 up regulated, 1 down regulated, and 2 with an unclear trend between Affymetrix and Febit data) that were regulated uniquely by day 12. One was down regulated only by day 19, and 13 were up regulated by day 26. Most of the 50 (31) miRNAs were already regulated by day 12 (see Venn diagram Figure 7).

**Figure 8:**
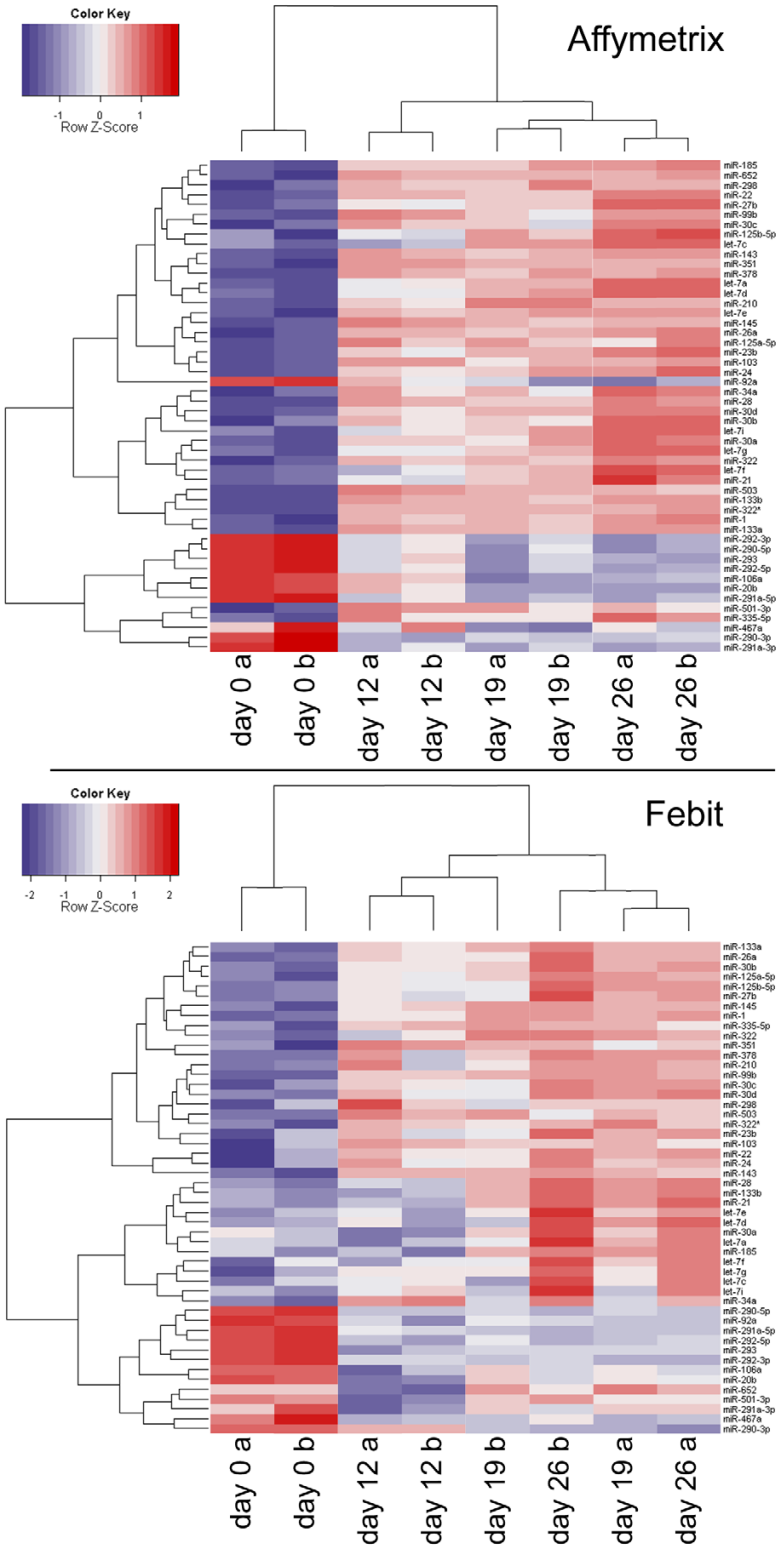
Heatmap of expression values of the 50 platform and pre-processing methods independent regulated miRNAs from Affymetrix platform (upper part) and Febit platform (lower part).

Clustering of miRNAs, which reacted similarly during cardiomyocyte differentiation, will help in the elucidation of the miRNA regulation pathway for cardiomyocyte differentiation. For this purpose, heat-maps were made for the expression values of the above-mentioned 50 miRNAs for data from both platforms (Figure 8). Columns in the upper part of figure 8 (for Affymetrix platform) showed that replicate samples were clustered together. Undifferentiated samples (day 0) were separated from differentiated samples (day 12, day 19 and day 26). Among differentiated samples, day 19 was closer to day 26 than to day 12. The sample cluster of Febit data (Figure 8 lower part) showed that replicates from day 19 and day 26 were mixed but day 0 samples and day 12 samples were well separated. The miRNAs were clustered into 2 groups in both heat-maps. The upper group was up regulated during the differentiation process while the lower group was down regulated in the differentiated samples.

### The selected 50 regulated miRNAs belong to cardiac differentiation and embryonic development-related groups

**Figure 9:**
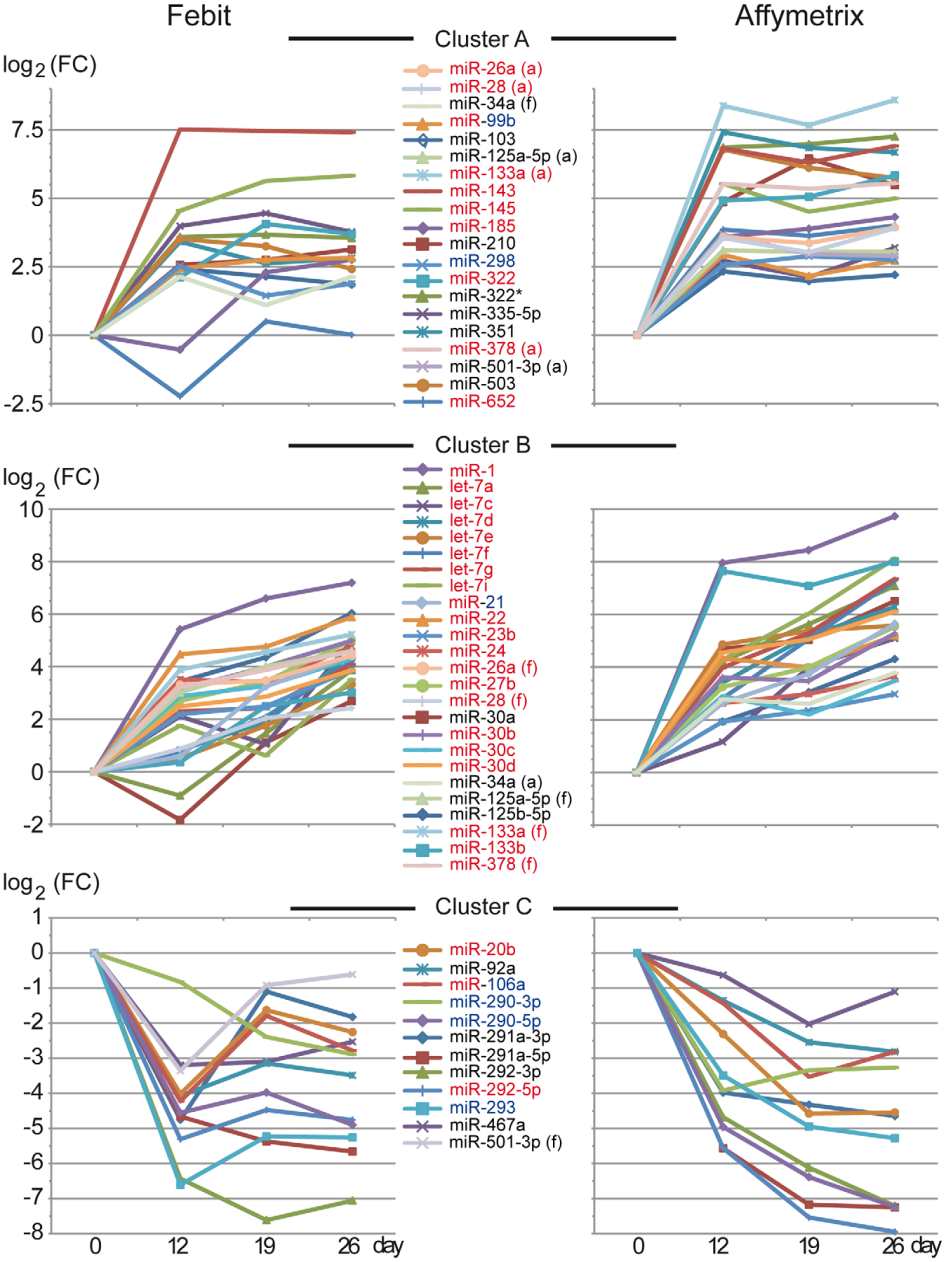
Changes in expression of selected miRNAs during time course of cardiomyocyte-specific differentiation/maturation. Three expression clusters of the 50 platform and pre-processing methods independent regulated miRNAs. (Cluster A) miRNAs which increased at day 12 and keep highly expressed afterwards, (Cluster B) miRNAs which incease continuously, (Cluster C) miRNAs which are down regulated during differentiation. miRNA names are colored in red for according to literature cardiac-related miRNAs and blue for according to literature ES cell related miRNAs. Y-axis represents log_2_ fold change over undifferentiated ES cells (day 0). miRNAs showing discrepancies in cluster membership dependent on array platform are marked with (a) for the Affymetrix and (f) for the febit result.

The time course of the miRNAs can tell us more about when and how the miRNAs were regulated during cardiac differentiation and maturation. For this reason the changes of expression values of miRNAs compared to undifferentiated samples have been illustrated in figure 9. Three major clusters were identified. Cluster A and cluster B were sub-groups of the up-regulated groups in the heat-map. Cluster A included miRNAs which increased until day 12, and then were maintained until day 26. Typical cardio-specific miRNAs (such as miR-143) could be found in this cluster, as well as some embryo-related miRNAs (such as miR-298) [25]. Some of the miRNAs in this cluster were reported to be involved in both cardiac- and embryonic-process. Cluster B included miRNAs which continually increased during the differentiation and maturation process. Most of the miRNAs in cluster B were cardiac differentiation related miRNAs, though there were also miRNAs reported as for both functions (e.g. miR-21). miRNAs in cluster C were down regulated after the beginning of differentiation. Many embryo miRNAs, such as miR-293, miR-290-3p or miR-290-5p were in cluster C. Cluster C also included miR-106a which is believed to be both ES cell-specific and cardiac-related [26].

### Target genes of differentiated expressed miRNAs are heart development-related

**Figure 10:**
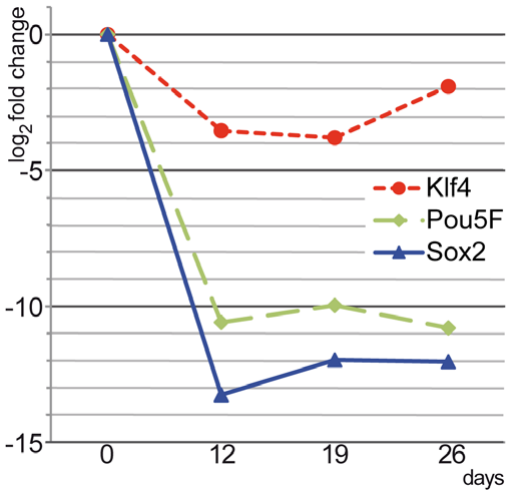
Embryonic marker genes are down regulated at day 12 of cardiomyocyte-specific differentiation. mRNA levels were measured at indicated timepoints using RT-qPCR. Relative gene expression of ES cell markers Klf4, Pou5F and Sox2 normalized to the expression of the reference gene GAPDH is shown. The results are expressed as fold change over day 0 values (undifferentiated ES cells). The y-axis represents log_2_ fold change relative to day 0.

miRNAs have been reported to be involved in the regulation process through interaction with mRNA. Target prediction makes it possible to speculate the regulation pathway of miRNA. In mouse gene ontology, 243 genes have been annotated under the term “GO:0007507: heart development”. 188 miRNAs have been predicted to be regulators for these genes. Among these 188 miRNAs, 23 were found in the 50 selected miRNAs. Six embryo marker genes (Myc, Sox2, Klf4, Lin28, Nanog, Pou5f1) were also predicted as regulators of miRNAs, 18 miRNAs were predicted to be target miRNAs (data not shown). As shown by RT-qPCR, the expression of the embryonic markers Klf4, Pou5F (Oct4) and Sox2 was completely down regulated by day 12 and remained depressed until day 26 (Figure 10). This effect was well synchronized with the up-regulation of miR-145.

## Discussion

miRNA in murine ES cells undergoing a cardiomyocyte-specific differentiation and maturation was profiled with the Affymetrix GeneChip platform and the Febit Geniom Realtime Analyzer. Instead of verifying miRNA profile results with RT-qPCR, the two independent high throughput platforms were used to verify results against each other. After differentiation analysis, miRNAs that were regulated in a platform-independent manner were identified for further study of their roles in cardiac differentiation and maturation.

### Expression level of miRNAs were distributed differently on the two platforms

Previous studies have shown that not all miRNAs are expressed in ES cells [15], [27]. Boxplot of the data from this study also showed that most of the miRNAs had low expression levels during the whole cardiomyocyte-specific differentiation and maturation process. There were more miRNAs detected in the differentiated samples than in the undifferentiated samples, based on the Febit platform results. However, this trend was not obvious for data from the Affymetrix platform. Since identical samples were applied in both studies, this could only be due to the different design principles and detection methods used by the platforms.

### Platforms and analysis methods have a strong influence on miRNA profiling results

In the PCA plots of the first 2 components, an absolute separation was observed in the axis of the first component, suggesting that the platforms exert a great influence on the profiling results. The Affymetrix data points were on the negative side while the Febit data points located on the positive side of the first principal component. This result indicates that the choice of platform may exert an even greater influence than the differentiation treatment itself. Along the axis of the second principal component sub-clusters were seen for sample from different stages during the maturation process especially for data generated using the Affymetrix platform. Day 0, which represents undifferentiated ES cells, is on one end of the axis, while day 12, day 19 and day 26 are on the other end of the axis. This cluster effect is also significant for data generated using the Febit platform, but day 0 was separated from other samples by less distance. The data for the day 19 and day 26 samples were difficult to separate on the Febit platform. This suggests that the Affymetrix platform may possess a higher sensitivity in detecting regulation of miRNA expression, an interpretation that was also supported by the differentiation analysis. Furthermore, according to the results of the 6 miRNAs analyzed by RT-qPCR, the Affymetrix miRNA array platform seemed to have a slight advantage over Febit miRNA array platform. Although a larger sample size would be required to allow a surer general statement regarding the reliability of the miRNA array platforms, it may be concluded that the results obtained by a single method should be considered with caution.

Analysis methods also have an influence on identifying differentially expressed miRNAs. In our study, the same samples analyzed on the same platform but only pre-processed with either RMA or VSN resulted in an approximately 30% variant hit list. For example, from the comparison of the results obtained with the Affymetrix platform from day 12 to day 0, we identified 124 miRNAs, which were significantly regulated after applying RMA and VSN methods. Of these miRNAs, 43 could only be identified after VSN while 48 miRNAs could be identified only after applying RMA. This result emphasized the importance of the verification procedure in miRNA profiling studies, especially when using microarray technology.

### miRNAs are differentially expressed during cardiac-specific differentiation and maturation

Despite the strong effect of platforms and pre-processing methods, there was a clear correlation between miRNA expression and cardiac-specific differentiation in the PCA plots. Furthermore, miRNA expression analysis also verified the separation of samples from undifferentiated ES cells and samples from Cor.At® cardiomyocytes. Fifty miRNAs were identified as being significantly regulated in a platform- and pre-processing independent manner. Compared to single high throughput platform analyses, the number of regulated miRNAs that were detected in the present study is relatively low. However, we believe that the application of 2 independent platforms for miRNA profiling results more plausible candidate groups. The small number of regulated miRNAs found in the present investigation could also be due to the use of pure populations of Cor.At® cardiomyocytes.

Thirty one of the 50 miRNAs had also been identified as regulated by day 12. This result indicates that the onset of the miRNAs changes during differentiation started before day 12. Among these 31 miRNAs, 18 were regulated throughout the whole differentiation and maturation period. An example of these 18 miRNAs is miR-22, which was recognized as one of the miRNAs, which increase dramatically during differentiation. Together with miR-21, miR-22 could regulate targets such as transforming growth factor-β-induced gene (TGFBi) [27], [28]. This indicates the possibility of negative regulation during differentiation. miR-291a-3p, miR-291a-5p, miR-292-3p, miR-290-5p and miR-293 belong to the miR-290-295 cluster [27]. This cluster of miRNAs decreases as ES cells differentiate. This result supports the hypothesis that ES-specific miRNAs were repressed during differentiation.

miR-21 has been intensively studied over recent years, in particular for its involvement in cardiovascular disease and cancer [29]. A study in 2007 [30] showed that miR-21 is up regulated in proliferating vascular smooth muscle cells (VSMCs) of the rat. This increase results in decreased cell proliferation and increased cell apoptosis in a dose-dependent manner in cultured rat aortic VSMCs. Another study found that miR-21 is strongly increased in failing heart [31], suggesting miR-21 to be an interesting target miRNA. Another example of up regulated miRNA is miR-145 which is significantly increased by day 12 and remains a high expression level until day 26. OCT4, SOX2 and KLF4 can be directly regulated by miR-145 [32]. As already known, OCT4, SOX2 and KLF4 are required for ES cell self-renewal and pluripotency. A high concentration of miR-145 is essential for stem cell differentiation. As expected, the RT-qPCR results confirmed that miR-145 target genes (Pou5F, Sox2 and Klf4) were down regulated by day 12 and remained at a low expression level during further cardiomyocyte maturation.

Two widely conserved miRNAs that display cardiac- and skeletal muscle–specific expression during development and in the adult are miR-1 and miR-133 [33], which are derived from a common precursor transcript (bicistronic) [34], [35]. miR-1 and miR-133a were up regulated in all three comparisons (days 12, 19, and 26) between Cor.At® cardiomyocytes and undifferentiated ES cells (day 0). In contrast, miR-206, which shares extensive sequence homology to miR-1, is found expressed exclusively in skeletal muscle with the co-transcribed miR-133b. Unsurprisingly, an increase of miR-206 was not observed during the present cardiomyocyte-specific differentiation process.

miR-1 has been reported to be abundant in rat heart but not in rat artery [36]. The expression of miR-1 was reported to be especially high in cardiac precursor cells [33], [35]. Experiments also revealed that excess miR-1 in the developing heart leads to a decreased pool of proliferating ventricular cardiomyocytes [35]. Many results suggest that miR-1 genes modulate the effects of critical cardiac regulatory proteins to control the balance between differentiation and proliferation during cardiogenesis. miR-1 could also be an important target for use in therapy of cardiovascular disease. miR-138, which has been reported to regulate cardiac patterning [28], was only observed to be up regulated in the Affymetrix data but not in the Febit data.

In the time course study of the 50 selected miRNAs, miR-145 showed the highest increase by day 12, and remained at a high expression level thereafter. This suggests that miR-145 (and the other miRNA members of cluster A, Figure 9) is not the candidate which triggers the maturation process of the Cor.At® cardiomyocytes during which gap-junctions are built. There are also miRNAs such as miR-1, miR-133a and miR-133b, which increased progressively at day 19 and day 26. Together with miR-21, which was first regulated after the cell-cell contact (i.e. day 12), they are good candidates to elucidate the maturation phase of cardiac-specific differentiation.

Many recent studies have already demonstrated that miRNAs are involved in critical biological processes. In this context, profiling miRNA expression won increasing emphasis. miRNAs were also believed to play an important role in ES cell differentiation and cardiovascular diseases. In the present manuscript paper, a transgenic mouse ES cell clone was used to generate uniform Cor.At® cardiomyocytes. miRNAs were profiled for undifferentiated transgenic ES cells (day 0) and at time points day 12, day 19 and day 26 during cardiomyocyte-specific differentiation and maturation using 2 high throughput microarray platforms provided from Affymetrix and Febit. Fifty miRNAs were identified as validly regulated from data generated from both platforms during cardiomyocyte-specific differentiation and maturation events. Although variation between platforms was high, the cross-validation procedure yields more reliable data. This provides a good basis for further research on the regulation of pathways in cardiac differentiation and maturation. On the other hand, our study demonstrates the necessity and efficiency of high throughput cross platform validation to minimize invalid results obtained by miRNA profiling with microarray platforms.

## Materials and Methods

### Cell culture

Mouse ES cells (D3, ATCC CRL 1934) were stably transfected with the α-MHC–Pac–IRES–EGFP vector containing the EGFP gene and the PuromycinR (Pac) cassette under control of the cardiac α-myosin heavy chain (α-MHC) promoter as previously described [22]. ES cells of a stably transfected clone (aPIG 44) were cultured on mouse embroynic fibroblasts in high-glucose Dulbecco's modified Eagle's medium (DMEM) supplemented with nonessential amino acids (0.1 mM), L-glutamine (2 mM), β-mercaptoethanol (0.1 mM), LIF (ESGR) (500 U/ml), neomycin (6 µg/mL), and batch-tested fetal calf serum (FCS) (15% v/v) (all Invitrogen, Karlsruhe, Germany).

To start differentiation, ES cells were trypsinized (day 0) and cultured in suspension on a horizontal shaker (GFL 3006, GFL, Braunschweig, Germany) to form embryoid bodies (EBs). Briefly, ES cells were transferred into Iscove's modified Dulbecco's Medium (IMDM) with 20% batch-tested FCS, nonessential amino acids (0.1 mM) and β-mercaptoethanol (0.1 mM) (Invitrogen, Karlsruhe, Germany) in a 10 cm bacterial Petri dish and cultured at 90 rpm, 37°C, 5% CO_2_, and 95% humidity for 48 h. At day 2, resulting EBs were transferred into 1000 mL spinner flasks (Integra Cell Spin, IBS, Fernwald, Germany) and cultured for additional 7 days at 37°C, 5% CO_2_, and 95% humidity. Medium was exchanged at day 5, day 7, and day 9; and Puromyicn (5 µg/mL) was added to select for cardiomyocytes at day 9. At day 12, remaining cardiobodies (embryoid bodies consisting of cardiomyocytes, Figure 1c) were trypsinized to obtain a single cell suspension of cardiomyocytes (Cor.At® cardiomyocytes) (Figure 1d).

6×10^6^ Cor.At® cardiomyocytes each were then seeded onto Fibronectin-coated 10 cm dishes (Becton Dickinson, Heidelberg, Germany) in IMDM medium as described above and cultured at 37°C, 5% CO_2_, and 95% humidity with daily media changes. All micorphotographs were taken using an Axiovert 100 M (Zeiss, Jena, Germany) equipped with a FITC filter set (AF Analysentechnik, Stuttgart, Germany).

### Immunostaining

For immunostaining, Cor.At® cardiomyocytes were seeded at 2×10^5^ cells/well on fibronectin-coated 24 well plates in IMDM 20% FCS and cultured as described above. At day 7 and day 14 after seeding (day 19 and day 26 after differentiation), cells were washed twice with PBS, fixed with 4% PFA for 30 minutes, permeabilized with 0.1% (w/v) Saponin in PBS for 30 minutes, and blocked for 1 h with 5% (w/v) BSA in PBS (all at RT). Between each of the following steps, cells were washed 3× using PBS with 0.8% (w/v) BSA and 0.1% (w/v) Saponin (PBS*).

Immunostaining for α-Actinin was performed by incubating the cells with a monoclonal anti-α-Actinin antibody (Sigma-Aldrich, Munich, Germany) diluted 1∶100 in PBS* at 4°C overnight, followed by incubation with a Cy2 conjugated goat-anti-mouse IgG anitbody (Sigma-Aldrich, Munich, Germany) diluted 1∶200 in PBS* for 1 h at 37°C.

Afterwards, the immunostaining for Connexin 43 was performed by incubating the pre-stained cells with a rabbit-anti-mouse Cx43 IgG antibody (Biotrend, Cologne, Germany) diluted 1∶200 in PBS* at 4°C overnight, followed by an incubation with a Cy3 conjugated goat-anti-rabbit IgG antibody (Dianova, Hamburg, Germany) diluted 1∶200 in PBS* for 1 h at 37°C. Cells were washed, and nuclei were stained with DAPI (1 mg/mL, Sigma-Aldrich, Munich, Germany) diluted in PBS* for 5 minutes at RT. After a final washing step, cells were covered with PBS and analyzed using an Axiovert 100 M (Zeiss, Jena, Germany) equipped with a Cy3 and a Cy2 filter set (AF Analysentechnik, Stuttgart, Germany).

### RNA isolation, quantification, and quality control

For day 0 RNA, ES cells were trypsinized at day -2, seeded onto a gelatin-coated 6 cm TC dish (Becton Dickinson, Heidelberg, Germany), and cultured for 48 h in DMEM 15% FCS as described above. The timing of this last passage was in parallel with the ES cell passage used to initiate differentiation.

For day 12 RNA, Cor.At® cells were seeded onto a Fibronectin coated 10 cm dish as described above and cultured for 6 h. The Cor.At® cardiomyocytes attached to the surface of the dish; remaining dead cells from the selection procedure were washed off before isolating day 12 RNA.

For all other days, RNA was prepared from the 10 cm dishes after washing the plates 2× with PBS. Total RNAs from cells after timepoints indicated were extracted by using PeqGold RNApure (Peqlab Biotechnology, Erlangen, Germany) according to the manufacturer's instructions. Quantity of total RNA was measured using a NanoDrop ND-1000 Spectrophotometer (NanoDrop Technologies, Wilmington, DE, USA). Optical density values at 260/280 were consistently above 1.9. The total RNA Quality was assayed on an Agilent BioAnalyzer (Agilent Technologies, Santa Clara, CA, USA). Only samples with intact, distinct ribosomal peaks were chosen for further analysis.

### RT-qPCR

#### Gene expression assays

First strand cDNA was reverse transcribed from 500 ng of total RNA using Superscript™II Reverse Transcriptase, Oligo (dT)12–18 and random hexamer primers according to the manufacturer's instructions (Invitrogen, Karlsruhe, Germany). Glycerinaldehyde-3-phosphate dehydrogenase (GAPDH), Krüppel like factor 4 (Klf4), Pou domain class 5 (Pou5f1/Oct4), and SRY-containg gene 2 (Sox2) were quantified using Individual TaqMan® Gene Expression Assays (Applied Biosystems, Foster City, CA, USA) on an ABI 7300 Real-Time PCR System. cDNA (25 ng) was mixed with TaqMan® Gene Expression Master Mix (Applied Biosystems, Foster City, CA, USA) and the appropriate TaqMan® Gene Expression Assays (Applied Biosystems, Foster City, CA, USA) for the gene of interest: (GAPDH - 4352932E, Klf4 - Mm00516104_m1, Pou5f1/Oct4 - Mm0353917_g1, Sox2 - Mm03053810_s1). To calculate relative gene expression we used the comparative quantification cycle (C_q_) method (2^−ΔΔCq^) [37]. All reactions were performed in triplicate and relative gene expression was normalized to the reference gene GAPDH. The results are expressed as fold change over day 0 values.

#### Micro RNA assays

Validation of expression of selected miRNAs was performed using commercially available pre-designed TaqMan RT-qPCR assays (Applied Biosystems, Foster City, CA, USA) using the same RNA samples as used for the microarray profiling. The TaqMan® MicroRNA Reverse Transcription Kit (Applied Biosystems, Foster City, CA, USA) was used for the preparation of cDNA. Reverse transcription reactions were performed in a volume of 20 µL at 16°C for 30 minutes, at 42°C for 30 minutes, and then terminated at 85°C for 5 minutes. Each reaction contained 20 ng of total RNA, and multiple (heptaplex) stem-loop miRNA-specific primers from the TaqMan MicroRNA Assays (each 12.5 nM), 2 mM dNTPs, 100 U MultiScribe™ Reverse Transcriptase, 1×Reverse Transcription buffer, and 5 U RNase Inhibitor. For qPCR of individual TaqMan MicroRNA Assays we used 0.75 µLTaqMan MicroRNA Assay Primer, 1.2 µL cDNA (10-fold diluted), 7.5 µL TaqMan 2× universal PCR master mix without UNG, and 5.55 µL nuclease-free water. All TaqMan MicroRNA Assays were run on an Applied Biosystems 7300 Real-Time PCR System with the following conditions: an initial step of 10 minutes at 95°C, followed by 40 cycles of 15 s at 95°C and 1 minute at 60°C. The assays used were: hsa-miR-208, hsa-miR-1, mmu-miR-292-3p, mmu-miR-295#, mmu-miR-501#, mmu-miR-715, and the small nucleolar RNA snoRNA202 as reference. To calculate relative miRNA expression we used the comparative quantification cycle (Cq) method (2^−ΔΔCq^) [37]. All reactions were performed in triplicate, and relative miRNA expression was normalized to expression of the reference small nucleolar RNA snoRNA202. Results are expressed as fold change over day 0 values. To illustrate the abundance of the miRNAs the Cq values before normalization are also presented.

### Affymetrix miRNA labelling, array hybridization and data pre-processing

Total RNA containing low molecular weight RNA was labelled using the Flashtag RNA labeling kit (Genisphere, Hatfield, PA, USA) according to the manufacturer's instructions. Briefly, for each sample, 2 µg total RNA were subjected a tailing reaction (2.5 mM MnCl_2_, ATP, Poly A Polymerase - incubation for 15 minutes at 37°) followed by ligation of the biotinylated signal molecule to the target RNA sample (1× Flash Tag ligation mix biotin, T4 DNA ligase - incubation for 30 minutes at RT) and adding of stop solution.

Each sample was hybridized to a GeneChip® miRNA Array (Affymetrix, Santa Clara, CA, USA) at 48°C and 60 rpm for 16 hours then washed and stained on Fluidics Station 450 (Fluidics script FS450_0003) and finally scanned on a GeneChip® Scanner 3000 7G (Affymetrix). The image data were analyzed with the miRNA QC Tool software for quality control (www.affymetrix.com/products_services/arrays/specific/mi_rna.affx#1_4). For each time point two independent experiments were performed under identical conditions. The expression values were summarized and normalized respectively with robust multi-array average (RMA) [38] and variance stabilization method (VSN) [39] using RMA and VSN packages in Bioconductor 2.5 under R 2.10 [40].

### Febit miRNA labelling, array hybridization and data pre-processing

The same samples were analyzed with a Geniom Realtime Analyzer (GRTA, Febit GmbH, Heidelberg, Germany) using the Geniom Biochip miRNA mus musculus. Each array contains 8 replicates of 710 miRNAs and miRNA star sequences as annotated in the Sanger miRBase 14.0. Sample labelling with biotin was carried out by microfluidic-based enzymatic on-chip labelling of miRNAs (MPEA) as described before [41]. Following hybridization for 16 hours at 42°C the biochip was washed automatically and a program for signal enhancement was processed with the GRTA. The resulting detection pictures were evaluated using the Geniom Wizard Software. For each array, the median signal intensity was extracted from the raw data file such that for each miRNA eight intensity values were calculated corresponding to each replicate copy of miRBase on the array. Following background correction, the eight replicate intensity values of each miRNA were summarized by their median value. To normalize the data across different arrays, quantile normalization and VSN were applied and all further analyses were carried out using the normalized and background subtracted intensity values.

### Bioinformatic analyses of Affymetrix and Febit miRNA microarray data

The overlapped miRNAs on both platforms were selected according to annotations provided from the corresponding chip manufacturer. The expression values of these overlapped miRNAs were then used for further analysis. Present/absent calls were made for both platforms. For Affymetrix detection, p-values were decided by Wilcoxon-test and Affymetrix-test; probe-sets with a p-value lower than 0.06 were called present. For Febit platform, a boolean value p is computed for every probe. This value indicates whether the respective miRNA is present or not. For a probe with intensity value *V*, the information if the probe is present is computed as follows: 
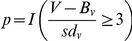
. Where *B_ν_* denotes the mean intensity of blank controls and *sd_ν_* indicates standard deviation of blank controls. Assuming that blank controls are normally distributed, the significance value for each probe would be 0.001.

Principle component analysis (PCA) was applied on normalized data from each of the platforms. The first 2 components of PCA were plotted using R [42]. Boxplots of the raw log_2_ intensities of both platforms as well as VSN normalized data were made using R function. Linear models were fitted for both datasets in order to distinguish differentially expressed miRNAs between different time points on the Affymetrix platform and the Febit platform, respectively (Linear Models for Microarray Data (LIMMA) Bioconductor package [43]). Comparisons were made between undifferentiated samples (day 0) and each of the differentiated samples (days 12, 19 and 26). The miRNAs, which showed adjusted p-value lower than 0.05, were selected as significantly differentially expressed. Venn diagrams and histograms for differential expression analysis results were made using R functions. Additionally, cluster analysis was made for the platform independent regulated miRNAs by using R functions. Target prediction of miRNAs was done using MIRror [44].

All array data are MIAME compliant. The raw data has been deposited in Gene Expression Omnibus (GEO) - Accession number: GSE24066.

## Supporting Information

Table S1 Complete list of all mouse miRNAs on both platforms; the miRNAs were categorized according to their present/absent call.
(DOCX)

## References

[1] Bhattacharya B, Puri S, Puri RK (2009). A review of gene expression profiling of human embryonic stem cell lines and their differentiated progeny.. Curr Stem Cell Res Ther.

[2] Glaser T, Schmandt T, Brustle O (2008). Generation and potential biomedical applications of embryonic stem cell-derived glial precursors.. J Neurol Sci.

[3] Sone M, Itoh H, Yamahara K, Yamashita JK, Yurugi-Kobayashi T (2007). Pathway for differentiation of human embryonic stem cells to vascular cell components and their potential for vascular regeneration.. Arterioscler Thromb Vasc Biol.

[4] Elaut G, Henkens T, Papeleu P, Snykers S, Vinken M (2006). Molecular mechanisms underlying the dedifferentiation process of isolated hepatocytes and their cultures.. Curr Drug Metab.

[5] Rowe C, Goldring CE, Kitteringham NR, Jenkins RE, Lane BS (2010). Network analysis of primary hepatocyte dedifferentiation using a shotgun proteomics approach.. J Proteome Res.

[6] Zhang Y, Nuglozeh E, Toure F, Schmidt AM, Vunjak-Novakovic G (2009). Controllable expansion of primary cardiomyocytes by reversible immortalization.. Hum Gene Ther.

[7] Katsumoto K, Shiraki N, Miki R, Kume S (2010). Embryonic and adult stem cell systems in mammals: ontology and regulation.. Dev Growth Differ.

[8] Enver T, Pera M, Peterson C, Andrews PW (2009). Stem cell states, fates, and the rules of attraction.. Cell Stem Cell.

[9] Murry CE, Keller G (2008). Differentiation of embryonic stem cells to clinically relevant populations: lessons from embryonic development.. Cell.

[10] Liang Y, Russell I, Walworth C, Chen C (2009). Gene expression in stem cells.. Crit Rev Eukaryot Gene Expr.

[11] Murray P, Edgar D (2004). The topographical regulation of embryonic stem cell differentiation.. Philos Trans R Soc Lond B Biol Sci.

[12] Jackson RJ, Hellen CU, Pestova TV (2010). The mechanism of eukaryotic translation initiation and principles of its regulation.. Nat Rev Mol Cell Biol.

[13] Minard ME, Jain AK, Barton MC (2009). Analysis of epigenetic alterations to chromatin during development.. Genesis.

[14] Hatfield S, Ruohola-Baker H (2008). microRNA and stem cell function.. Cell Tissue Res.

[15] Wang Y, Russell I, Chen C (2009). MicroRNA and stem cell regulation.. Curr Opin Mol Ther.

[16] Yin JQ, Zhao RC, Morris KV (2008). Profiling microRNA expression with microarrays.. Trends Biotechnol.

[17] Barone AD, Beecher JE, Bury PA, Chen C, Doede T (2001). Photolithographic synthesis of high-density oligonucleotide probe arrays.. Nucleosides Nucleotides Nucleic Acids.

[18] Puceat M (2008). Protocols for cardiac differentiation of embryonic stem cells.. Methods.

[19] Zeineddine D, Papadimou E, Mery A, Menard C, Puceat M (2005). Cardiac commitment of embryonic stem cells for myocardial repair.. Methods Mol Med.

[20] Doetschman TC, Eistetter H, Katz M, Schmidt W, Kemler R (1985). The in vitro development of blastocyst-derived embryonic stem cell lines: formation of visceral yolk sac, blood islands and myocardium.. J Embryol Exp Morphol.

[21] Kolossov E, Lu Z, Drobinskaya I, Gassanov N, Duan Y (2005). Identification and characterization of embryonic stem cell-derived pacemaker and atrial cardiomyocytes.. FASEB J.

[22] Kolossov E, Bostani T, Roell W, Breitbach M, Pillekamp F (2006). Engraftment of engineered ES cell-derived cardiomyocytes but not BM cells restores contractile function to the infarcted myocardium.. J Exp Med.

[23] Kolossov E, Fleischmann BK, Liu Q, Bloch W, Viatchenko-Karpinski S (1998). Functional characteristics of ES cell-derived cardiac precursor cells identified by tissue-specific expression of the green fluorescent protein.. J Cell Biol.

[24] Sachinidis A, Fleischmann BK, Kolossov E, Wartenberg M, Sauer H (2003). Cardiac specific differentiation of mouse embryonic stem cells.. Cardiovasc Res.

[25] Tang F, Hajkova P, Barton SC, Lao K, Surani MA (2006). MicroRNA expression profiling of single whole embryonic stem cells.. Nucleic Acids Res.

[26] Foshay KM, Gallicano GI (2009). miR-17 family miRNAs are expressed during early mammalian development and regulate stem cell differentiation.. Dev Biol.

[27] Houbaviy HB, Murray MF, Sharp PA (2003). Embryonic stem cell-specific MicroRNAs.. Dev Cell.

[28] Cordes KR, Srivastava D, Ivey KN (2010). MicroRNAs in cardiac development.. Pediatr Cardiol.

[29] Krichevsky AM, Gabriely G (2009). miR-21: a small multi-faceted RNA.. J Cell Mol Med.

[30] Ji R, Cheng Y, Yue J, Yang J, Liu X (2007). MicroRNA expression signature and antisense-mediated depletion reveal an essential role of MicroRNA in vascular neointimal lesion formation.. Circ Res.

[31] Thum T, Gross C, Fiedler J, Fischer T, Kissler S (2008). MicroRNA-21 contributes to myocardial disease by stimulating MAP kinase signalling in fibroblasts.. Nature.

[32] Xu N, Papagiannakopoulos T, Pan G, Thomson JA, Kosik KS (2009). MicroRNA-145 regulates OCT4, SOX2, and KLF4 and represses pluripotency in human embryonic stem cells.. Cell.

[33] Ivey KN, Muth A, Arnold J, King FW, Yeh RF (2008). MicroRNA regulation of cell lineages in mouse and human embryonic stem cells.. Cell Stem Cell.

[34] Chen JF, Mandel EM, Thomson JM, Wu Q, Callis TE (2006). The role of microRNA-1 and microRNA-133 in skeletal muscle proliferation and differentiation.. Nat Genet.

[35] Zhao Y, Samal E, Srivastava D (2005). Serum response factor regulates a muscle-specific microRNA that targets Hand2 during cardiogenesis.. Nature.

[36] Zhang C (2008). MicroRNAs: role in cardiovascular biology and disease.. Clin Sci (Lond).

[37] Livak KJ, Schmittgen TD (2001). Analysis of relative gene expression data using real-time quantitative PCR and the 2(-Delta Delta C(T)) Method.. Methods.

[38] Irizarry RA, Bolstad BM, Collin F, Cope LM, Hobbs B (2003). Summaries of Affymetrix GeneChip probe level data.. Nucleic Acids Res.

[39] Huber W, von Heydebreck A, Sultmann H, Poustka A, Vingron M (2002). Variance stabilization applied to microarray data calibration and to the quantification of differential expression.. Bioinformatics.

[40] Gentleman RC, Carey VJ, Bates DM, Bolstad B, Dettling M (2004). Bioconductor: open software development for computational biology and bioinformatics.. Genome Biol.

[41] Vorwerk S, Ganter K, Cheng Y, Hoheisel J, Stahler PF (2008). Microfluidic-based enzymatic on-chip labeling of miRNAs.. N Biotechnol.

[42] Computing RFfS, Team RDC (2008). R: A Language and Environment for Statistical Computing;.

[43] Smyth G (2002). Statistical applications in genetics and molecular biology.

[44] Friedman Y, Naamati G, Linial M (2010). MiRror: a combinatorial analysis web tool for ensembles of microRNAs and their targets.. Bioinformatics.

