# Supplementary material for: MicroRNA Profiling during Cardiomyocyte-Specific Differentiation of Murine Embryonic Stem Cells Based on Two Different miRNA Array Platforms
Source: PLoS One. 2011 Oct 3;6(10):e25809. doi: 10.1371/journal.pone.0025809 (PMC3185008; doi:10.1371/journal.pone.0025809)
Supplement: Table S1 — Complete list of all mouse miRNAs on both platforms; the miRNAs were categorized according to their present/absent call. (DOCX) [file pone.0025809.s001.docx]

| **at least one present on both platforms (Affymetrix & Febit)** | | | | | | | |
| --- | --- | --- | --- | --- | --- | --- | --- |
| miR-1 | miR-30d | miR-181d | miR-320 | miR-450b-3p | miR-503 | miR-700 | miR-22* |
| let-7a | miR-30e | miR-182 | miR-322 | miR-450a-5p | miR-504 | miR-702 | miR-24-1* |
| let-7b | miR-31 | miR-185 | miR-324-3p | miR-452 | miR-532-3p | miR-704 | miR-24-2* |
| let-7c | miR-34a | miR-191 | miR-324-5p | miR-455 | miR-532-5p | miR-705 | miR-28* |
| let-7d | miR-34b-3p | miR-193 | miR-328 | miR-466a-3p | miR-541 | miR-706 | miR-30a* |
| let-7e | miR-92a | miR-193b | miR-329 | miR-466b-3p | miR-542-3p | miR-709 | miR-30e* |
| let-7f | miR-92b | miR-194 | miR-330 | miR-466b-3-3p | miR-542-5p | miR-710 | miR-34c* |
| let-7g | miR-93 | miR-195 | miR-335-5p | miR-466c-3p | miR-652 | miR-711 | miR-106b* |
| let-7i | miR-98 | miR-197 | miR-338-5p | miR-466c-5p | miR-574-3p | miR-713 | miR-124* |
| miR-15b | miR-99a | miR-200b | miR-339-5p | miR-466d-3p | miR-574-5p | miR-714 | miR-127* |
| miR-15a | miR-99b | miR-203 | miR-341 | miR-466e-3p | miR-598 | miR-720 | miR-133a* |
| miR-16 | miR-100 | miR-207 | miR-342-3p | miR-466f | miR-666-5p | miR-721 | miR-138* |
| miR-17 | miR-101b | miR-208a | miR-346 | miR-466f-3p | miR-667 | miR-744 | miR-140* |
| miR-18a | miR-103 | miR-208b | miR-350 | miR-466f-5p | miR-668 | miR-759 | miR-141* |
| miR-18b | miR-106a | miR-210 | miR-351 | miR-466g | miR-669a | miR-760 | miR-181a-1* |
| miR-19b | miR-106b | miR-212 | miR-361 | miR-466h | miR-669b | miR-762 | miR-297a* |
| miR-20a | miR-107 | miR-214 | miR-362-5p | miR-466i | miR-669c | miR-763 | miR-297c* |
| miR-20b | miR-127 | miR-218 | miR-363 | miR-466j | miR-669d | miR-805 | miR-299* |
| miR-21 | miR-128 | miR-221 | miR-370 | miR-467a | miR-669e | miR-874 | miR-302a* |
| miR-22 | miR-125a-5p | miR-290-5p | miR-378 | miR-467c | miR-669f | miR-875-3p | miR-322* |
| miR-23a | miR-125b-5p | miR-291a-3p | miR-379 | miR-467e | miR-669h-3p | miR-877 | miR-330* |
| miR-23b | miR-129-3p | miR-291a-5p | miR-380-5p | miR-467f | miR-669h-5p | miR-878-5p | miR-378* |
| miR-24 | miR-130a | miR-292-3p | miR-382 | miR-467g | miR-669i | miR-1187 | miR-431* |
| miR-25 | miR-130b | miR-292-5p | miR-383 | miR-468 | miR-669k | miR-1188 | miR-467a* |
| miR-26a | miR-133a | miR-293 | miR-409-5p | miR-469 | miR-672 | miR-1190 | miR-467b* |
| miR-26b | miR-133b | miR-294 | miR-410 | miR-483 | miR-673-5p | miR-1192 | miR-467d* |
| miR-27a | miR-143 | miR-295 | miR-411 | miR-484 | miR-674 | miR-1195 | miR-467e* |
| miR-27b | miR-145 | miR-296-3p | miR-412 | miR-486 | miR-675-3p | miR-1196 | miR-483* |
| miR-28 | miR-148a | miR-296-5p | miR-423-3p | miR-491 | miR-675-5p | miR-1224 | miR-485* |
| miR-29a | miR-148b | miR-297a | miR-423-5p | miR-493 | miR-676 | miR-7a* | miR-503* |
| miR-29b | miR-151-5p | miR-297b-3p | miR-425 | miR-494 | miR-678 | let-7f* | miR-674* |
| miR-29c | miR-152 | miR-298 | miR-429 | miR-495 | miR-689 | miR-17* | miR-676* |
| miR-30a | miR-154 | miR-301a | miR-431 | miR-497 | miR-690 | miR-18a* | miR-712* |
| miR-30b | miR-181a | miR-301b | miR-433 | miR-499 | miR-691 | miR-20b* | miR-744* |
| miR-30c | miR-181c | miR-302d | miR-434-3p | miR-500 | miR-696 | miR-21* | miR-877* |
| **on both platforms both all absent (Affymetrix & Febit)** | | | | | | | |
| miR-9 | miR-184 | miR-367 | miR-471 | miR-683 | miR-764-3p | miR-9* | miR-300* |
| miR-10a | miR-186 | miR-375 | miR-496 | miR-684 | miR-764-5p | miR-10a* | miR-376a* |
| miR-10b | miR-188-3p | miR-376c | miR-505 | miR-687 | miR-802 | miR-10b* | miR-376b* |
| miR-32 | miR-190 | miR-384-5p | miR-511 | miR-692 | miR-804 | miR-19a* | miR-382* |
| miR-33 | miR-196a | miR-448 | miR-509-5p | miR-693-3p | miR-873 | miR-26b* | miR-455* |
| miR-34b-5p | miR-202-3p | miR-449a | miR-540-5p | miR-693-5p | miR-876-3p | miR-29a* | miR-463* |
| miR-96 | miR-202-5p | miR-449b | miR-544 | miR-694 | miR-876-5p | miR-29c* | miR-470* |
| miR-105 | miR-204 | miR-451 | miR-546 | miR-697 | miR-879 | miR-101a* | miR-488* |
| miR-135a | miR-219 | miR-453 | miR-568 | miR-701 | miR-880 | miR-181a-2* | miR-708* |
| miR-135b | miR-220 | miR-463 | miR-590-3p | miR-707 | miR-881 | miR-186* | miR-742* |
| miR-137 | miR-291b-3p | miR-464 | miR-590-5p | miR-717 | miR-882 | miR-196a* | miR-879* |
| miR-139-3p | miR-297b-5p | miR-465a-3p | miR-654-5p | miR-741 | miR-883a-3p | miR-200a* |  |
| miR-142-3p | miR-302b | miR-465a-5p | miR-669g | miR-743a | miR-883a-5p | miR-200c* |  |
| miR-144 | miR-302c | miR-465c-5p | miR-673-3p | miR-743b-3p | miR-1194 | miR-203* |  |
| miR-147 | miR-340-3p | miR-466e-5p | miR-677 | miR-743b-5p | miR-1199 | miR-214* |  |
| miR-153 | miR-343 | miR-467d | miR-680 | miR-758 | let-7a* | miR-218-1* |  |
| **only Affymetrix all absent** | | | | | | | |
| miR-1-2-as | miR-211 | miR-362-3p | miR-466a-5p | miR-592 | miR-688 | miR-1191 | miR-374* |
| miR-7b | miR-216a | miR-365 | miR-466b-5p | miR-654-3p | miR-695 | miR-1197 | miR-376c* |
| miR-19a | miR-216b | miR-369-3p | miR-466d-5p | miR-666-3p | miR-715 | let-7c-2* | miR-881* |
| miR-101a | miR-217 | miR-384-3p | miR-466k | miR-669j | miR-718 | let-7i* |  |
| miR-126-5p | miR-297c | miR-449c | miR-467h | miR-679 | miR-719 | miR-15b* |  |
| miR-136 | miR-323-5p | miR-450b-5p | miR-547 | miR-681 | miR-761 | miR-136* |  |
| miR-190b | miR-338-3p | miR-465b-5p | miR-551b | miR-682 | miR-878-3p | miR-145* |  |
| miR-196b | miR-339-3p | miR-465c-3p | miR-582-5p | miR-686 | miR-1186 | miR-146b* |  |
| **only Febit all absent** | | | | | | | |
| miR-7a | miR-151-3p | miR-290-3p | miR-369-5p | miR-490 | miR-712 | miR-27b* | miR-199b* |
| miR-34c | miR-155 | miR-291b-5p | miR-374 | miR-501-3p | miR-742 | miR-29b* | miR-200b* |
| miR-122 | miR-181b | miR-299 | miR-376a | miR-501-5p | miR-770-3p | miR-30b* | miR-218-2* |
| miR-124 | miR-183 | miR-300 | miR-376b | miR-509-3p | miR-770-5p | miR-30c-1* | miR-293* |
| miR-125a-3p | miR-187 | miR-302a | miR-377 | miR-539 | miR-871 | miR-30c-2* | miR-294* |
| miR-125b-3p | miR-188-5p | miR-323-3p | miR-380-3p | miR-540-3p | miR-872 | miR-31* | miR-295* |
| miR-126-3p | miR-192 | miR-325 | miR-381 | miR-543 | miR-875-5p | miR-33* | miR-302b* |
| miR-129-5p | miR-199a-3p | miR-326 | miR-409-3p | miR-582-3p | miR-883b-3p | miR-92a* | miR-302c* |
| miR-132 | miR-199a-5p | miR-327 | miR-421 | miR-615-3p | miR-883b-5p | miR-93* | miR-325* |
| miR-134 | miR-199b | miR-331-3p | miR-434-5p | miR-615-5p | miR-1193 | miR-99b* | miR-411* |
| miR-138 | miR-200a | miR-331-5p | miR-450a-3p | miR-653 | miR-1198 | miR-125b* | miR-425* |
| miR-139-5p | miR-200c | miR-335-3p | miR-465b-3p | miR-665 | let-7b* | miR-130b* | miR-433* |
| miR-140 | miR-201 | miR-337-3p | miR-466l | miR-670 | let-7c-1* | miR-135a* | miR-872* |
| miR-141 | miR-205 | miR-337-5p | miR-467b | miR-671-3p | let-7d* | miR-148a* |  |
| miR-142-5p | miR-206 | miR-340-5p | miR-470 | miR-671-5p | let-7g* | miR-150* |  |
| miR-146a | miR-215 | miR-342-5p | miR-485 | miR-685 | miR-15a* | miR-154* |  |
| miR-146b | miR-222 | miR-344 | miR-487b | miR-698 | miR-16* | miR-183* |  |
| miR-149 | miR-223 | miR-345-3p | miR-488 | miR-703 | miR-20a* | miR-191* |  |
| miR-150 | miR-224 | miR-345-5p | miR-489 | miR-708 | miR-27a* | miR-193* |  |
| **only on Affymetrix miRNA array - Affymetrix at least one present** | | | | | | | |
| miR-699 |  |  |  |  |  |  |  |
| **only on Febit miRNA chip - Febit at least one present** | | | | | | | |
| miR-669l | miR-1897-3p | miR-1906 | miR-1937a | miR-1946a | miR-1961 | miR-2135 | miR-2146 |
| miR-669m | miR-1898 | miR-1907 | miR-1937b | miR-1946b | miR-1962 | miR-2137 | miR-2182 |
| miR-669n | miR-1899 | miR-1929 | miR-1937c | miR-1947 | miR-1967 | miR-2138 | miR-1982* |
| miR-669o | miR-1900 | miR-1931 | miR-1939 | miR-1948 | miR-1969 | miR-2139 |  |
| miR-1274a | miR-1901 | miR-1933-3p | miR-1940 | miR-1953 | miR-2132 | miR-2142 |  |
| miR-1839-3p | miR-1902 | miR-1933-5p | miR-1944 | miR-1957 | miR-2133 | miR-2143 |  |
| miR-1839-5p | miR-1903 | miR-1935 | miR-1945 | miR-1959 | miR-2134 | miR-2145 |  |
| **only on Febit miRNA chip - Febit all absent** | | | | | | | |
| miR-432 | miR-1897-3p | miR-1927 | miR-1941-3p | miR-1952 | miR-1964 | miR-1982.1 | miR-2183 |
| miR-599 | miR-1898 | miR-1928 | miR-1941-5p | miR-1954 | miR-1965 | miR-1982.2 |  |
| miR-664 | miR-1899 | miR-1930 | miR-1942 | miR-1955 | miR-1966 | miR-1983 |  |
| miR-767 | miR-1900 | miR-1932 | miR-1943 | miR-1956 | miR-1968 | miR-2136 |  |
| miR-1306 | miR-1901 | miR-1934 | miR-1949 | miR-1958 | miR-1970 | miR-2140 |  |
| miR-1893 | miR-1902 | miR-1936 | miR-1950 | miR-1960 | miR-1971 | miR-2141 |  |
| miR-1896 | miR-1903 | miR-1938 | miR-1951 | miR-1963 | miR-1981 | miR-2144 |  |

**Table S1:** Complete list of all mouse miRNAs on both platforms; the miRNAs were categorized according to their present/absent call.
